# Knowledge and Perceptions of Human Papillomavirus (HPV) Vaccination Among Parents of Pediatric Patients at Nnamdi Azikiwe University Teaching Hospital

**DOI:** 10.7759/cureus.100627

**Published:** 2026-01-02

**Authors:** CFC Ogbuefi, Paul Ofoegbu, Chukwudi Jesse Nwajagu, K E Ogbuefi, J O Egbunike, O L Ezika

**Affiliations:** 1 Family Medicine, Federal University Teaching Hospital, Owerri, NGA; 2 Community Medicine, Nnamdi Azikiwe University Teaching Hospital, Nnewi, NGA; 3 Family Medicine, Federal Medical Centre, Abuja, NGA

**Keywords:** hpv, knowledge, nnewi, parents, pediatric patients, perception, vaccine

## Abstract

Background

Human papillomavirus (HPV) is the most common sexually transmitted infection globally and the primary cause of cervical cancer. Despite the availability of safe and effective vaccines, HPV vaccination uptake remains low in Nigeria. Understanding parental knowledge and perceptions is crucial for improving vaccination rates.

Objective

This study aimed to assess the knowledge and perceptions of HPV vaccination among parents of pediatric patients at Nnamdi Azikiwe University Teaching Hospital (NAUTH), Nnewi, Nigeria.

Materials and methods

A descriptive cross-sectional study was conducted among 384 parents of pediatric patients attending NAUTH between May and June 2025. Data were collected using pre-tested, interviewer-administered structured questionnaires. Knowledge and perception scores were calculated and categorized. Data analysis was performed using IBM SPSS Statistics for Windows, Version 26.0 (Released 2018; IBM Corp., Armonk, NY, USA), with chi-square tests used to assess associations between sociodemographic variables and knowledge/perception levels. Statistical significance was set at p < 0.05.

Results

The mean age of respondents was 35.8 ± 7.2 years, with 67.4% being female. Most of the respondents (58.6%) had a tertiary education. Overall, 62.8% of the respondents had heard of HPV, while only 31.5% had heard of HPV vaccination. The knowledge scores revealed that 45.3% had poor knowledge, 38.5% had fair knowledge, and 16.2% had good knowledge of HPV vaccination. Perception analysis showed that only 52.3% had negative perceptions, 31.5% had neutral perceptions, and 16.2% had positive perceptions toward HPV vaccination. Higher education level (p = 0.001) and healthcare worker status (p < 0.001) were significantly associated with better knowledge, as only 8.3% of the respondents' children had received HPV vaccination.

Conclusions

While knowledge and perceptions of HPV vaccination among parents at NAUTH were poor, respondents were more willing to have their children vaccinated. Educational interventions targeting parents, particularly those with lower education levels, are urgently needed to improve HPV vaccination uptake and ultimately reduce cervical cancer burden in Nigeria.

## Introduction

Human papillomavirus (HPV) is the most commonly sexually transmitted infection worldwide, with over 200 identified types, of which approximately 40 affect the anogenital region [1]. The high-risk HPV types, particularly types 16 and 18, are responsible for approximately 70% of cervical cancer cases globally [2]. Cervical cancer is the fourth most common malignancy among women worldwide, with an estimated 660,000 new cases and 350,000 deaths in 2022 [3].

The burden of cervical cancer is disproportionately higher in low- and middle-income countries (LMICs), including Nigeria, where limited screening programs and low vaccination coverage contribute to the higher incidence as well as the mortality rates [4]. In Nigeria alone, cervical cancer is the second most common malignancy among women, with an age-standardized incidence rate of 18.4 per 100,000 and approximately 12,075 new cases annually [5].

HPV vaccines have been available since 2006 and have demonstrated remarkable efficacy in preventing HPV infections and their associated diseases [6]. Three vaccines are currently licensed globally: the bivalent vaccine (HPV types 16 and 18), the quadrivalent vaccine (HPV types 6, 11, 16, and 18), and the nonavalent vaccine (HPV types 6, 11, 16, 18, 31, 33, 45, 52, and 58) [7]. The World Health Organization (WHO) recommends HPV vaccination for girls aged 9-14 years as part of the comprehensive cervical cancer prevention strategies [8].

Despite global recommendations and proven vaccine efficacy, HPV vaccination coverage remains low in many African countries, including Nigeria [9]. The Nigerian government introduced HPV vaccination into the national immunization program in 2022, targeting girls aged 9-14 years, but the vaccine uptake has been limited [10]. Nigeria’s national immunization program currently uses the quadrivalent HPV vaccine, which protects against HPV types 6, 11, 16, and 18, for routine vaccination of eligible girls. Multiple factors contribute to low vaccination rates, including limited awareness, poor access to vaccines, cultural beliefs, and of course negative perceptions among parents and caregivers [11].

Parental knowledge and attitudes play a critical role in vaccination decisions, as parents are the primary decision-makers for their children’s healthcare [12]. Previous studies in Nigeria have documented varying levels of HPV knowledge among different populations, with generally low awareness of HPV vaccination [13,14]. Understanding parental knowledge and perceptions is not only essential but crucial for designing effective educational interventions and public health strategies to improve vaccination uptake.

This study was designed to assess the knowledge and perceptions of HPV vaccination among parents of pediatric patients at Nnamdi Azikiwe University Teaching Hospital (NAUTH), Nnewi, Nigeria, in order to identify gaps and inform targeted interventions for improving vaccination coverage.

## Materials and methods

Study design and setting

This was a descriptive cross-sectional study conducted at the pediatric outpatient clinics of Nnamdi Azikiwe University Teaching Hospital (NAUTH), Nnewi, Anambra State, Nigeria. NAUTH is a tertiary healthcare facility that serves as a major referral center for southeastern Nigeria. The study was conducted between May and June 2025.

Study population

The study population comprised parents or primary caregivers of pediatric patients attending the pediatric outpatient clinics at NAUTH. Parents or caregivers who provided informed consent and had at least one child aged 0-18 years were eligible for inclusion. Although the national HPV vaccination program targets girls aged 9-14 years, parents of children aged 0-18 years were included to reflect real-world clinic attendance patterns, where parents often bring multiple children of different ages, and to capture parental knowledge and perceptions that influence vaccination decisions for all eligible daughters in the household. Exclusion criteria included severely ill parents or caregivers who could not participate, those who declined consent, and parents or caregivers of critically ill children requiring immediate medical attention. For this study, primary caregivers were defined as adults who were responsible for making healthcare decisions for the child and who accompanied the child to the clinic visit (e.g., parents, grandparents, or legal guardians).

Sample size determination

The sample size was calculated using the Cochran formula for cross-sectional studies:

$n = \frac{z^{2} \times p \times \left(1 - p\right)}{d^{2}}$

where *n* = required sample size, *Z* = Z-score corresponding to 95% confidence level (1.96), *p* = estimated proportion of parents with knowledge of HPV vaccination (50% or 0.5, assuming maximum variability), and *d* = desired precision or margin of error (5% or 0.05):

$n = \frac{(1.96)^{2} \times 0.5 \times 0.5}{(0.05)^{2}}$

$n = \frac{3.8416 \times 0.25}{0.0025}$

$n = \frac{0.9604}{0.0025}$

$n = 384.16$

The minimum sample size was calculated as 384 respondents. After accounting for a 10% non-response rate, the final sample size was adjusted to 422 respondents.

Sampling technique

A systematic random sampling method was employed to select study participants. The average daily attendance at the pediatric outpatient clinics was approximately 60 patients. Over the two-month study period (approximately 50 clinic days), the expected total attendance was 3,000 parents/caregivers. The sampling interval (k) was calculated as:

$k = \frac{\text{population size}}{\text{sample size}}$

$k = \frac{3000}{422}$

$k = 7$

The first participant was randomly selected from the first seven parents, and subsequently, every seventh parent attending the clinic was approached for recruitment until the desired sample size was achieved.

Data collection instrument

Data were collected using a pre-tested, interviewer-administered structured questionnaire adapted from previous studies on HPV knowledge and vaccination attitudes [15,16]. The questionnaire comprised four main sections: (1)* sociodemographic characteristics *(age, gender, marital status, educational level, occupation, religion, ethnicity, and number of children); (2) *awareness and knowledge of HPV infection and HPV vaccination* (questions assessing prior exposure to information about HPV and HPV vaccines); (3) *perceptions of HPV vaccination and willingness to vaccinate *(questions covering beliefs about vaccine safety, necessity, religious concerns, cultural acceptability, and willingness to vaccinate children); and (4) *factors affecting HPV vaccination knowledge and uptake *(11 statements assessing financial, access, cultural and informational barriers).

The full questionnaire, including all sociodemographic, knowledge, perception, and influencing-factor items with response options, is provided in Appendix 1. The instrument was developed by the investigators and adapted, with contextual modifications, from previously published HPV vaccination knowledge and attitude surveys conducted in Nigeria and other settings.

Section B (Appendix 1) comprised 22 items assessing awareness and factual knowledge of HPV infection and HPV vaccination. Of these, 15 items directly assessing factual knowledge were included in the composite knowledge score, while the remaining items assessed awareness and sources of information and were analyzed descriptively. Each correct response was assigned a score of 1, while incorrect or “I don’t know” responses were assigned a score of 0, yielding a total possible knowledge score ranging from 0 to 15.

Knowledge levels were classified using modified Bloom’s cutoff criteria, a widely accepted method in knowledge, attitude, and practice studies. Scores were categorized as poor knowledge (<60% of the total score; 0-8), fair knowledge (60%-79%; 9-11), and good knowledge (≥80%; 12-15), as commonly applied in KAP studies [15,16].

Section C (Appendix 1) utilized a five-point Likert scale (strongly disagree = 1, disagree = 2, neutral = 3, agree = 4, strongly agree = 5). Negatively framed statements were reverse-scored. The total perception score ranged from 12 to 60, categorized as negative (12-28), neutral (29-44), and positive (45-60) perceptions.

The questionnaire was pre-tested on 40 parents at a nearby health facility not included in the main study, and necessary modifications were made based on feedback to ensure clarity and comprehension.

Data collection procedure

Four trained research assistants with backgrounds in public health conducted the interviews. Training covered study objectives, ethical considerations, questionnaire administration techniques, and data quality assurance. Parents or caregivers were approached in the waiting areas of the pediatric outpatient clinics, and the study purpose was explained. After obtaining informed consent, face-to-face interviews lasting approximately 15-20 minutes were conducted in English or Igbo, depending on the respondent's preference. The questionnaire was developed and administered in English, which is the official language of instruction and clinical communication in Nigeria. For respondents who required clarification of specific terms, trained bilingual research assistants provided on-the-spot explanations in Igbo while maintaining the original meaning of the item.

Ethical considerations

Ethical approval was obtained from the NAUTH Research Ethics Committee (Reference Number: NAUTH/CS/66/Vol.17/VER.3/66/2025/60) prior to study commencement. Written informed consent was obtained from all participants after explaining the study purpose, the voluntary nature of participation, confidentiality measures, and the right to withdraw at any time without consequences. No personal identifiers were recorded, and all data were stored securely.

Data analysis

Data were entered into Microsoft Excel 2019 (Microsoft Corp., Redmond, WA, USA) and analyzed using IBM SPSS Statistics for Windows, Version 26.0 (Released 2018; IBM Corp., Armonk, NY, USA). Descriptive statistics, including frequencies, percentages, means, and standard deviations, were calculated for sociodemographic variables, knowledge scores, and perception scores. Categorical variables were presented as frequencies and percentages, while continuous variables were presented as means with standard deviations.

Associations between categorical variables (e.g., sociodemographic characteristics, knowledge category, and perception category) were assessed using chi-square tests, with Fisher’s exact tests applied when expected cell counts were less than five. Test statistics (χ^2^) with corresponding p-values are reported for key associations. Independent samples t-tests were used to compare mean knowledge and perception scores across binary sociodemographic variables.

For key associations of interest (education level and healthcare worker status with knowledge and perception), odds ratios (ORs) with 95% confidence intervals (CIs) were calculated using 2 × 2 contingency tables after collapsing knowledge into good versus not-good and perception into positive versus non-positive categories. Statistical significance was set at p < 0.05 with 95% CIs.

## Results

Sociodemographic characteristics of respondents

A total of 384 respondents completed the study, yielding a 100% response rate (Table 1). The mean age of respondents was 35.8 ± 7.2 years, with the majority (198, 51.6%) in the 30-39 years age group. Female respondents predominated (259, 67.4%), reflecting the typical pattern of mothers being primary caregivers. Most respondents were married (328, 85.4%), had a tertiary education (225, 58.6%), and were Christians (362, 94.3%) (Table 1). Nearly half (178, 46.4%) had 3-4 children. Only 52 (13.5%) of respondents were healthcare workers.

**Table 1:** Sociodemographic characteristics of respondents (N = 384)

Variable	Frequency (n)	Percentage (%)
Age group (years)		
20-29	76	19.8
30-39	198	51.6
40-49	89	23.2
50 and above	21	5.5
Mean age ± SD	35.8 ± 7.2	
Gender		
Male	125	32.6
Female	259	67.4
Marital status		
Single	34	8.9
Married	328	85.4
Divorced/separated	14	3.6
Widowed	8	2.1
Educational level		
No formal education	18	4.7
Primary education	45	11.7
Secondary education	96	25.0
Tertiary education	225	58.6
Occupation		
Unemployed	62	16.1
Unskilled worker	48	12.5
Skilled worker	89	23.2
Business/trading	108	28.1
Professional/civil servant	77	20.1
Healthcare worker		
Yes	52	13.5
No	332	86.5
Religion		
Christianity	362	94.3
Islam	18	4.7
Traditional	4	1.0
Number of children		
1-2	156	40.6
3-4	178	46.4
5 and above	50	13.0

Awareness of HPV and HPV vaccination

Respondent awareness of HPV and HPV vaccination is summarized in Table 2. While 241 (62.8%) had heard of HPV, only 121 (31.5%) had heard of HPV vaccination. Healthcare workers were the most common information source (58, 47.9%), followed by mass media (32, 26.4%). Only 32 (8.3%) of respondents’ children had received HPV vaccination.

**Table 2:** Awareness of HPV and HPV vaccination among respondents (N = 384) HPV: human papillomavirus.

Awareness variable	Frequency (n)	Percentage (%)
Ever heard of HPV		
Yes	241	62.8
No	143	37.2
Ever heard of HPV vaccination		
Yes	121	31.5
No	263	68.5
Source of information (n=121)		
Healthcare worker	58	47.9
Mass media (TV/radio)	32	26.4
Internet/social media	18	14.9
Friends/family	13	10.7
Child vaccinated against HPV		
Yes	32	8.3
No	352	91.7

Knowledge of HPV and HPV vaccination

Knowledge was the highest for basic facts, such as HPV being a sexually transmitted infection (198, 51.6%) and its association with cervical cancer (176, 45.8%) (Table 3). However, based on the modified Bloom’s cutoff criteria, knowledge about specific vaccination details was lower, with only 98 (25.5%) knowing the recommended age group and 76 (19.8%) knowing the correct dosing schedule.

**Table 3:** Distribution of correct responses to HPV knowledge questions (N = 384) HPV: human papillomavirus.

Knowledge item	Correct (n)	Percentage (%)
HPV is a sexually transmitted infection	198	51.6
HPV causes cervical cancer	176	45.8
HPV affects both males and females	145	37.8
HPV vaccine is available	134	34.9
HPV vaccine prevents cervical cancer	128	33.3
HPV vaccine is recommended for girls aged 9-14 years	98	25.5
HPV vaccine can be given to boys	87	22.7
HPV vaccine requires two doses for optimal protection	76	19.8
HPV vaccine is safe	112	29.2
HPV vaccine does not cause infertility	89	23.2
Vaccination before sexual debut is most effective	108	28.1
HPV vaccine is available in Nigeria	67	17.4
HPV vaccine is part of routine immunization	54	14.1
Vaccinated individuals still need cervical screening	94	24.5
HPV vaccine protects against genital warts	82	21.4

The overall distribution of HPV vaccination knowledge levels is shown in Table 4. The knowledge score was 6.2 ± 3.8 out of a possible 15 points. Overall, 174 (45.3%) of respondents had poor knowledge, 148 (38.5%) had fair knowledge, and only 62 (16.2%) had good knowledge of HPV vaccination.

**Table 4:** Distribution of HPV vaccination knowledge levels based on modified Bloom’s cutoff among parents of pediatric patients at NAUTH, Nnewi (N = 384) Knowledge levels were derived from 15 core factual knowledge items selected from the 22-item questionnaire (Section B, Appendix 1) and categorized using modified Bloom’s cutoff criteria. HPV: human papillomavirus, NAUTH: Nnamdi Azikiwe University Teaching Hospital.

Knowledge level	Frequency (n)	Percentage (%)
Poor (0-8 points)	174	45.3
Fair (9-11 points)	148	38.5
Good (12-15 points )	62	16.2
Mean knowledge score ± SD	6.2 ± 3.8	

Perceptions and attitudes toward HPV vaccination

The majority of respondents (302, 78.7%) expressed a need for more information about HPV vaccination. Concerns about side effects were common (214, 55.7% agreed or strongly agreed). Relatively few respondents believed HPV vaccination conflicted with religion (30, 7.8%) or culture (41, 10.7%), though many were neutral on these issues.

**Figure 1:**
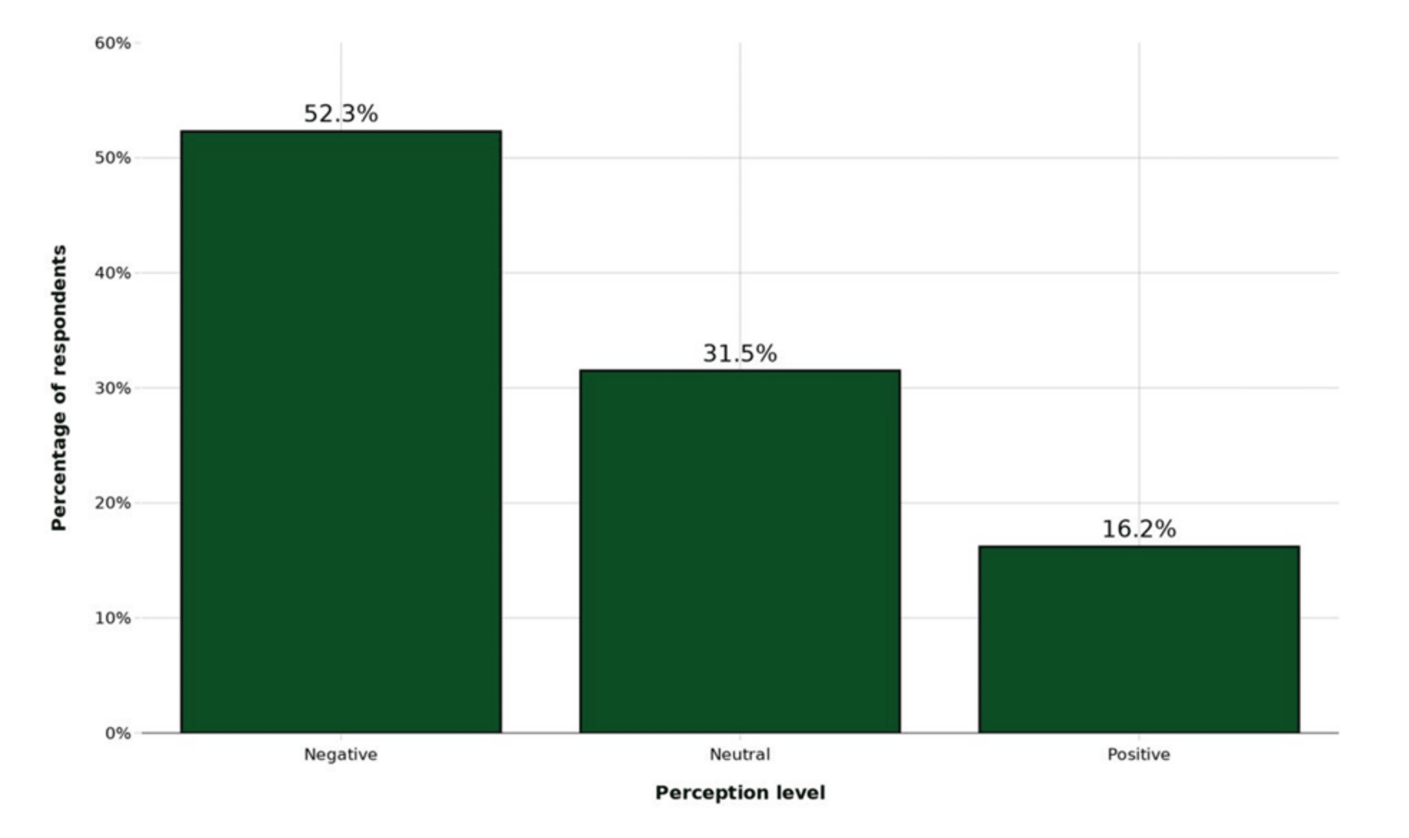
Distribution of perceptions toward HPV vaccination among parents of pediatric patients at NAUTH, Nnewi (N = 384). HPV: human papillomavirus, NAUTH: Nnamdi Azikiwe University Teaching Hospital.

The distribution of perception levels toward HPV vaccination is illustrated in Figure 1. The mean perception score was 30.4 ± 9.6 out of a possible 60 points. Overall, 201 (52.3%) had negative perceptions, 121 (31.5%) had neutral perceptions, and 62 (16.2%) had positive perceptions toward HPV vaccination.

Association between sociodemographic characteristics and knowledge

Educational level was significantly associated with knowledge level (χ^2^ = 38.71, p = 0.001). Respondents with tertiary education had higher odds of good knowledge compared with those with lower education (OR 3.94, 95% CI 1.98-7.84) (Table 5). Healthcare workers had significantly better knowledge than non-healthcare workers (χ^2^ = 19.49, p < 0.001), with 34.6% of healthcare workers having good knowledge compared to 13.3% of non-healthcare workers (OR 3.47, 95% CI 1.80-6.66). Age, gender, and marital status showed no significant association with knowledge levels.

**Table 5:** Association between sociodemographic characteristics and knowledge levels Test statistic from Pearson's chi-square test; p< 0.05 considered statistically significant.

Variable	Poor	Fair	Good	Test statistic	p-value
	n (%)	n (%)	n (%)	(χ^2^)	
Age group					
20-29 years	38 (50.0)	28 (36.8)	10 (13.2)	2.46	0.156
30-39 years	85 (42.9)	79 (39.9)	34 (17.2)		
40-49 years	42 (47.2)	34 (38.2)	13 (14.6)		
50+ years	9 (42.9)	7 (33.3)	5 (23.8)		
Gender					
Male	62 (49.6)	45 (36.0)	18 (14.4)	1.41	0.423
Female	112 (43.2)	103 (39.8)	44 (17.0)		
Educational level					
No formal/primary	42 (66.7)	18 (28.6)	3 (4.8)	38.71	0.001
Secondary	56 (58.3)	32 (33.3)	8 (8.3)		
Tertiary	76 (33.8)	98 (43.6)	51 (22.7)		
Healthcare worker					
Yes	12 (23.1)	22 (42.3)	18 (34.6)	19.49	<0.001
No	162 (48.8)	126 (38.0)	44 (13.3)		
Marital status					
Single	18 (52.9)	11 (32.4)	5 (14.7)	1.02	0.612
Married	146 (44.5)	128 (39.0)	54 (16.5)		
Others	10 (45.5)	9 (40.9)	3 (13.6)		

Association between sociodemographic characteristics and perceptions

Educational level was significantly associated with perception level (p = 0.002), with tertiary-educated respondents having higher odds of positive perceptions (OR 3.94, 95% CI 1.98-7.84) compared to those with lower education (Table 6). Healthcare workers had significantly more positive perceptions (30.8%) compared to non-healthcare workers (13.9%) (p = 0.003; OR 2.76, 95% CI 1.42-5.38) (Table 7). There was a strong, significant association between knowledge level and perception level (p < 0.001), with respondents having good knowledge demonstrating predominantly positive perceptions (30, 48.4%), while those with poor knowledge had predominantly negative perceptions (118, 67.8%).

**Table 6:** Odds ratios and 95% confidence intervals for predictors of good knowledge and positive perception ORs and 95% CIs calculated from a 2 x 2 contingency table; p-values from χ^2^ test.

Predictor	Outcome	OR (95% CI)	p-value
Higher vs lower education	Good vs not-good knowledge	3.94 (1.98-7.84)	<0.001
Healthcare worker vs others	Good vs not-good knowledge	3.47 (1.8-6.66)	<0.001
Higher vs lower education	Positive vs non-positive perception	3.94 (1.98-7.84 )	0.002
Healthcare worker vs others	Positive vs non-positive perception	2.76 (1.42-5.38)	0.003

**Table 7:** Association between sociodemographic characteristics and perception levels Test statistic from Pearson's chi-square test; p < 0.05 considered statistically significant.

Variable	Negative	Neutral	Positive	Test statistic	p-value
	n (%)	n (%)	n (%)	(χ^2^)	
Age group					
20-29 years	42 (55.3)	24 (31.6)	10 (13.2)	0.96	0.845
30-39 years	101 (51.0)	63 (31.8)	34 (17.2)		
40-49 years	47 (52.8)	27 (30.3)	15 (16.9)		
50+ years	11 (52.4)	7 (33.3)	3 (14.3)		
Gender					
Male	68 (54.4)	39 (31.2)	18 (14.4)	2.03	0.721
Female	133 (51.4)	82 (31.7)	44 (17.0)		
Educational level					
No formal/primary	45 (71.4)	15 (23.8)	3 (4.8)	29.08	0.002
Secondary	62 (64.6)	26 (27.1)	8 (8.3)		
Tertiary	94 (41.8)	80 (35.6)	51 (22.7)		
Healthcare worker					
Yes	18 (34.6)	18 (34.6)	16 (30.8)	6.09	0.003
No	183 (55.1)	103 (31.0)	46 (13.9)		
Knowledge level					
Poor	118 (67.8)	46 (26.4)	10 (5.7)	99.76	<0.001
Fair	72 (48.6)	54 (36.5)	22 (14.9)		
Good	11 (17.7)	21 (33.9)	30 (48.4)		

## Discussion

This study assessed knowledge and perceptions of HPV vaccination among parents of pediatric patients at NAUTH, Nnewi. The findings revealed suboptimal knowledge and predominantly negative perceptions toward HPV vaccination, with significant associations between educational level, healthcare worker status, and both knowledge and perception levels.

Awareness of HPV and HPV vaccination

The finding that 62.8% had heard of HPV but only 31.5% knew of HPV vaccination is higher than some previous Nigerian studies (41.5% and 18.8%, respectively) [17,18], likely reflecting increased public health campaigns. However, the gap between HPV and vaccination awareness suggests general knowledge does not translate to prevention awareness.

Low vaccination awareness (31.5%) parallels other sub-Saharan African countries (Kenya, 28.4%; Uganda, 35.2%) [19,20], highlighting the need for targeted vaccination campaigns. Healthcare workers, as the primary information source (47.9%), underscore their critical role in parent counseling during clinical encounters.

Knowledge of HPV vaccination

The mean knowledge score of 6.2 ± 3.8 (41.3%), with only 16.2% demonstrating good knowledge, mirrors findings from Lagos (22.1% good knowledge) and Benin City (58.3% poor knowledge) [21,22]. General HPV knowledge was highest (transmission, 51.6%; cervical cancer association, 45.8%), but vaccination-specific knowledge was low (recommended age, 25.5%; dosing schedule, 19.8%). Only 17.4% knew vaccines were available in Nigeria despite the introduction of the 2022 program [10], indicating insufficient communication about accessibility.

Perceptions and attitudes toward HPV vaccination

Negative perceptions (52.3%) predominated, with a mean score of 30.4 ± 9.6 (50.7%). Safety concerns (55.7%) and infertility myths (30.8%) were major barriers documented in other African studies [23-25]. Religious (7.8%) and cultural (10.7%) conflicts were less prominent. Notably, 78.7% sought more information, indicating openness to evidence-based education.

Sociodemographic factors associated with knowledge and perceptions

Educational level significantly predicted both knowledge and perceptions. Tertiary-educated respondents had better knowledge (22.7%) and perceptions (22.7%) versus primary/no education (4.8% each). Healthcare workers had superior knowledge (34.6% vs 13.3%) and perceptions (30.8% vs 13.9%), though only one-third achieved good knowledge (p < 0.001; p = 0.003). Strong knowledge-perception association (p < 0.001) with good-knowledge respondents showing 48.4% positive perceptions versus 67.8% negative for poor-knowledge respondents supports knowledge improvement as an intervention pathway. Age, gender, and marital status showed no significant associations.

HPV vaccination coverage

Vaccination coverage (8.3%) was far below WHO's 90% target, driven by low awareness, poor knowledge, negative perceptions, and systemic barriers. While comparable to Nigeria's national rate, it lags far behind countries with established programs (70%-80%). This knowledge-behavior gap underscores that awareness alone is insufficient without addressing perceptions and access.

Implications for public health interventions

The findings have several important implications for improving HPV vaccination uptake in Nigeria.

Targeted Educational Campaigns

Mass education campaigns using multiple channels (healthcare facilities, mass media, social media, schools, religious institutions) should provide accurate, culturally appropriate information about HPV, cervical cancer, and vaccination benefits and safety.

Healthcare Provider Training

Healthcare workers require comprehensive training on HPV vaccination to effectively counsel parents and address concerns. Given that healthcare workers were the primary information source, enhancing their knowledge and communication skills could have multiplicative effects.

Addressing Safety Concerns

Proactive communication about vaccine safety, including data from millions of vaccinated individuals globally and surveillance systems monitoring adverse events, should be prioritized to counter misinformation.

Dispelling Myths

Specific attention to common misconceptions, particularly regarding infertility and sexual behavior, is essential. Evidence-based messages addressing these myths should be incorporated into all educational materials.

School-Based Vaccination Programs

Implementing school-based HPV vaccination programs could improve access and coverage, particularly in areas where healthcare facility access is limited.

Community Engagement

Engaging community and religious leaders as vaccine champions could help address cultural and religious concerns and increase acceptance.

Improving Vaccine Accessibility

Ensuring consistent vaccine supply, reducing costs through government subsidies or free vaccination programs, and expanding vaccination sites would address structural barriers.

Strengths and limitations

Strengths

Adequate sample size, high response rate, validated instruments, and systematic sampling enhance reliability. Assessment of both knowledge and perceptions provides a comprehensive understanding of vaccination decision factors.

Limitations

Cross-sectional design precludes causal inference. A single-center location may limit generalizability. Social desirability bias may affect responses. The study did not assess actual access barriers. Future longitudinal and qualitative studies would strengthen findings.

## Conclusions

Knowledge and perceptions of HPV vaccination among parents of pediatric patients at NAUTH, Nnewi, were generally suboptimal, although many parents expressed willingness to have their children vaccinated. Higher educational level and healthcare worker status were associated with more favorable knowledge and perceptions, highlighting the influence of educational and professional factors on vaccine acceptance. Strengthened provider-based counseling, improved access to the quadrivalent HPV vaccine within Nigeria’s national immunization program, and targeted educational interventions for parents with lower education levels are needed to enhance HPV vaccination uptake and reduce the burden of cervical cancer in this setting.

## Appendices

Appendix 1

This questionnaire was developed by the investigators for this study and adapted, with contextual modifications, from previously published HPV vaccination knowledge and attitude surveys and studies that applied modified Bloom’s cut-off criteria for knowledge classification [15,16].

Section A: Sociodemographic Characteristics

**Table 8:** Sociodemographic characteristics

Item	Question	Response options
1	Age at last birthday (years)	Open response (numeric)
2	Gender	a) Male b) Female
3	Marital status	a) Single b) Married c) Divorced d) Separated e) Widowed
4	Religion	a) Christianity b) Islam African traditional religion c) Atheist d) Others
5	ethnicity	a) Igbo b) Yoruba c) Hausa d) Others
6	Highest educational level	a) No formal education b) Primary c) Secondary d) Tertiary e) Postgraduate
7	occupation	a) Unemployed b) Business c) Artisan d) Civil servant e) Healthcare worker
8	Number of children	a) 0 b) 1 c) 2 d) 3 or more
9	Do you have any daughters	a) Yes b) No
10	If yes what are their ages	a) 9-14 years b) 15-19 years c) 20+ years
11	Average monthly family income (naira)	a) <50,000 b) 100,000-150,000 c) 150,000-200,000

Section B: Knowledge of HPV Infection and Vaccination

**Table 9:** Knowledge of HPV infection and vaccination

Item	Question	Response options
12	Have you heard of HPV infection?	a) Yes b) No
13	Source of HPV information	a) Self-study b) Mass media c) Health professional d) School e) Friends/peers f) Health-related institution g) Others
14	HPV affects both males and females	a) Yes b) No c) I don’t know
15	HPV causes cervical cancer	a) Yes b) No c) I don’t know
16	HPV causes genital warts	a)Yes b) No c) I don’t know
17	HPV is transmitted through	a) Sexual intercourse b) Blood contact c) Handshake d) Shared objects e) I don’t know
18	Many sexual partners increase HPV risk	a) Yes b) No c) I don’t know
19	Prevention of HPV infection	a) Abstinence b) Avoid blood contact c) Protected sex d) I don’t know
20	HPV infection can be cured with antibiotics	a)Yes b) No c) I don’t know
21	HPV always has visible symptoms	a) Yes b) No c) I don’t know
22	Have you heard of HPV vaccination?	a) Yes b) No
23	Source of HPV vaccine information	a) Self-study b) Mass media c) Health professional d) School e) Friends/relatives f) Health-related institution
24	HPV vaccine available in Nigeria	a) Yes b) No
25	Where can HPV vaccine be obtained?	a) Health centers b) Hospitals c) Outreach stations d) I don’t know
26	Recommended number of HPV doses	a) 1 b) 2 c) 3 d) Others
27	Recommended age for HPV vaccination	a)9–14 years b) Above 14 years c) Others d) I don’t know
28	Most effective before sexual debut	a) Yes b) No c) I don’t know
29	Protects against most cervical cancers	a) Yes b) No c) I don’t know
30	Protects against all STIs	a) Yes b) No c) I don’t know
31	Screening still important after vaccination	a) Yes b) No c) I don’t know
32	Does not protect against all viruses	a) Yes b) No c) I don’t know
33	Risk of cervical cancer remains after vaccination	a) Yes b) No c) I don’t know

Section C: Perception of HPV Vaccination and Willingness to Vaccinate

**Table 10:** Perception and willingness

Item	Question	Response options
34	Importance of HPV vaccination	a) Very important b) Somewhat important c) Not very important d) Not at all important
35	HPV vaccine is safe	a) Yes b) No c) Not sure
36	Approve child receiving HPV vaccine	a) Yes b) No c) Not sure
37	Has your daughter been vaccinated?	a) Yes b) No
38	Reason for vaccination	a) Physician recommendation b) Protection from future infection c) Close contact with cervical cancer
39	Reason for not vaccinating	a) Insufficient info b) Child too young c) Vaccine is new d) Safety concerns e) No sexual activity f) Not recommended g) Others
40	Plan to vaccinate in the future	a) Yes b) No c) Not sure
41	Recommend vaccine to others	a) Yes b) No c) Not sure
42	Religion permits vaccination	a) Yes b) No c) Not sure
43	Risk of HPV high without vaccine	a) Yes b) No c) I don’t know
44	Belief forbids vaccination	a) Yes b) No c) I don’t know

Section D: Factors Affecting the Knowledge and Perception of HPV Vaccination

**Table 11:** Factors affecting the knowledge and perception of HPV vaccination

S/N	Statement	Agree	Disagree	I don't know
45	Finance is a major barrier to the uptake of HPV vaccine			
46	Level of education can affect the use of HPV vaccination			
47	Distance to vaccination site is a barrier to HPV vaccination			
48	Misconception about HPV vaccination			
49	The belief that the child is too young to take the vaccine			
50	Concern about the adverse effects			
51	My religion does not permit me to take the vaccine			
52	My culture is against HPV vaccination			
53	Fear of promiscuity in the vaccinated girl			
54	Inadequate knowledge or information about the vaccine			
55	Time interval between each dose is too long			
